# Bioactive Phytochemicals from Wild *Arbutus unedo* L. Berries from Different Locations in Portugal: Quantification of Lipophilic Components

**DOI:** 10.3390/ijms160614194

**Published:** 2015-06-23

**Authors:** Daniela F. S. Fonseca, Ângelo C. Salvador, Sónia A. O. Santos, Carla Vilela, Carmen S. R. Freire, Armando J. D. Silvestre, Sílvia M. Rocha

**Affiliations:** 1 Organic Chemistry, Natural and Agro-Food Products (QOPNA), Department of Chemistry, University of Aveiro, 3810-193 Aveiro, Portugal; E-Mails: danielafonseca@ua.pt (D.F.S.F.); angelomcsalvador@ua.pt (A.C.S.); 2 Aveiro Institute of Materials (CICECO), Department of Chemistry, University of Aveiro, 3810-193 Aveiro, Portugal; E-Mails: santos.sonia@ua.pt (S.A.O.S.); cvilela@ua.pt (C.V.); cfreire@ua.pt (C.S.R.F.)

**Keywords:** *Arbutus unedo* L., ripe berries, GC–MS analysis, lipophilic composition, triterpenoids, fatty acids, sterols

## Abstract

The lipophilic composition of wild *Arbutus unedo* L. berries, collected from six locations in Penacova (center of Portugal), as well as some general chemical parameters, namely total soluble solids, pH, titratable acidity, total phenolic content and antioxidant activity was studied in detail to better understand its potential as a source of bioactive compounds. The chemical composition of the lipophilic extracts, focused on the fatty acids, triterpenoids, sterols, long chain aliphatic alcohols and tocopherols, was investigated by gas chromatography–mass spectrometry (GC–MS) analysis of the dichloromethane extracts. The lipophilic extractives of the ripe *A. unedo* berries ranged from 0.72% to 1.66% (*w*/*w* of dry weight), and consisted mainly of triterpenoids, fatty acids and sterols. Minor amounts of long chain aliphatic alcohols and tocopherols were also identified. Forty-one compounds were identified and among these, ursolic acid, lupeol, α-amyrin, linoleic and α-linolenic acids, and β-sitosterol were highlighted as the major components. To the best of our knowledge the current research study provides the most detailed phytochemical repository for the lipophilic composition of *A. unedo*, and offers valuable information for future valuation and exploitation of these berries.

## 1. Introduction

*Arbutus unedo* L. (*A. unedo*), also known as strawberry tree, is a Mediterranean plant belonging to the Ericaceae family and Vaccionioideae (or Arbutoideae) subfamily, with an extension along the Atlantic coast of Europe [1,2]. *A. unedo* is an evergreen shrub with spherical berries covered with conical hair-like spikes, soft yellow pulp and an average diameter of 2 cm [2,3]. *A. unedo* berries exploitation can be traced back to ancient times where their use in folk medicine is claimed, namely in treatment of kidney diseases, cardiovascular, gastrointestinal, dermatologic and urological disorders [4,5]. Nowadays, the market associated to these berries is mainly oriented to the production of alcoholic distillates and also for jellies and jams [6], and are seldom eaten as fresh fruits [7,8].

Understanding the chemical composition of foods, particularly focusing on the phytochemical fingerprinting, has become central to research in order to address the increasing demand for nutraceuticals in foods [9]. The chemical composition of strawberry tree berries regarding the macro- and micro-nutrients and phenolic compounds is relatively well known [6,7,8,10,11,12,13,14,15,16,17,18,19,20,21,22,23,24,25]. In the lipophilic fraction, the presence of polyunsaturated fatty acids, namely α-linolenic (C18:3 ^(Δ9, 12, 15)^) and linoleic acid (C18:2 ^(Δ9, 12)^) [12,15,18,24], of triterpenoids, such as lupeol, α- and β-amyrin, olean-l2-en-3β,23-diol, lupenone, amyrone, ursolic aldehyde, α-amyrenone, uvaol, and ursolic and oleanolic acids [26,27] and of sterols, such as 5-α-cholestane, cholestan-3-one, cholesterol, stigmasterol and stigmast-4-en-3-one [27] has been reported. However, to the best of our knowledge, there is a lack of studies regarding the detailed quantification of these lipophilic components in *A. unedo* berries.

The particular interest in fatty acids, triterpenoids and sterols, results from their recognized benefits in human nutrition and therefore their importance for the valorization of these berries: lipophilic components have shown a wide range of biological activities such as hypocholesterolemic effect, anti-inflammatory, antitumor activities among others [28,29].

In this context, the present study aims to increase the depth of knowledge about the lipophilic fraction (focused on the fatty acids, long chain aliphatic alcohols, triterpenoids, sterols and tocopherols) by GC–MS of wild *A. unedo* berries lipophilic extracts. In order to establish a more representative lipophilic profile, wild samples harvested from six locations in Penacova (center of Portugal) were evaluated. Moreover, to contribute to a more complete characterization of *A. unedo* berries, this study also provides baseline information concerning total soluble solids, pH, titratable acidity, total phenolic content and antioxidant activity.

## 2. Results and Discussion

### 2.1. General Chemical Characterization

The general chemical parameters of the ripe *A. unedo* berries, namely total soluble solids (TSS), titratable acidity (TA), pH, total phenolic content (TPC), antioxidant activity and water content are displayed in Table 1. The GPS coordinates and the dichloromethane extraction yields are also presented.

In the present study, *A. unedo* berries revealed water content ranging between 61.13% in location 3 and 69.80% in location 5. These values were comparable to the previously reported for these berries which ranges from 42.78% to 72.59% [22,30]. The TSS of *A. unedo* berries ranged from 22.4 ± 0.3 to 30.1 ± 0.8 °Brix. Fruits from location 2 and 3 presented the lowest and the highest values, respectively. The TSS of *A. unedo* berries was similar to previously reported data from Portuguese fruits (≈22 °Brix) [20] and from other European origins (16.50–31.68 °Brix) [30,31,32]. The TA of the fruits collected from the six locations was very similar, ranging between *ca.* 0.8 g of malic acid per 100 g fresh weight in location 4, and *ca.* 0.9 g of malic acid per 100 g fresh weight, in location 6. TA for Portuguese fruits was assessed for the first time and the results were within the range reported for the fruits harvested out of Portugal (0.4–1.59 g of malic acid per 100 g fresh weight) [21,30,31,32]. Similarly to TA, pH of the Portuguese fruits was evaluated here for the first time and ranged from 2.97 ± 0.05 in location 2, to 3.17 ± 0.07 in location 4. pH values of the berries under study were lower than the ones already reported for *A. unedo* fruits, ranging between 3.21 and 4.6 [21,22,32]. TPC, expressed as mg gallic acid per 100 g fresh weight, varied from 1160 ± 13 in location 2, to 2222 ± 35, in location 6. TPC in *A. unedo* berries reached higher values than those already published, not only considering Portuguese samples (≈445–941 mg of gallic acid per 100 g fresh weight), but also the European ones (590–1973 mg of gallic acid per 100 g fresh weight) [22,30].

The antioxidant activity of the studied *A. unedo* berries ranged between 0.278 ± 0.02, in location 6, and 0.589 ± 0.01 mg of extract per mL DPPH, in location 2. The fruits collected from location 6 presented the lowest EC_50_ value, revealing the highest radical scavenging activity among the samples and therefore, the highest antioxidant activity. The results obtained were within the range reported in literature for these ripe berries (0.25–0.79 mg extract per mL DPPH) [15,18,33].

The comparison between the fruits collected from the six locations in the center of Portugal (Table 1) allowed us to observe a wide variability in the chemical parameters investigated. The differences detected can be attributed to various factors such as year, genetic variability (genetic data are not available, however different morphological characteristics of the berries and leaves may support this idea) and geographic position [17,22].

**Table 1:** Geographical (GPS) coordinates, general chemical characterization and dichloromethane extraction yields of *A. unedo* berries harvested from six locations in Penacova (center of Portugal).

Location	GPS Coordinates			Moisture (%)	Total Soluble Solids ^a^	Titratable Acidity ^b^	pH	Total Phenolic Content ^c^	Antioxidant Activity ^d^	Extraction Yield (%, *w*/*w*)
	Latitude	Longitude	Altitude (m)							
1	40°18ʹ22.3ʹʹ N	8°10ʹ00.5ʹʹ W	200.76	69.80	24.4 ± 0.5	0.89 ± 0.02	3.08 ± 0.02	1342 ± 76	0.469 ± 0.02	1.66
2	40°17ʹ33.2ʹʹ N	8°11ʹ52.1ʹʹ W	125.00	62.53	22.4 ± 0.3	0.88 ± 0.02	2.97 ± 0.05	1160 ± 13	0.589 ± 0.01	1.56
3	40°17ʹ36.7ʹʹ N	8°11ʹ51.1ʹʹ W	123.22	61.13	30.1 ± 0.8	0.85 ± 0.09	3.17 ± 0.04	1185 ± 18	0.359 ± 0.03	1.48
4	40°16ʹ53.4ʹʹ N	8°12ʹ25.8ʹʹ W	100.00	61.87	25.7 ± 0.6	0.81 ± 0.10	3.17 ± 0.07	1889 ± 66	0.356 ± 0.01	1.58
5	40°16ʹ37.4ʹʹ N	8°11ʹ57.5ʹʹ W	75.00	67.87	28.3 ± 0.5	0.89 ± 0.09	3.09 ± 0.07	2183 ± 48	0.343 ± 0.06	1.55
6	40°16ʹ34.7ʹʹ N	8°11ʹ50.3ʹʹ W	75.00	69.47	22.9 ± 0.2	0.91 ± 0.02	3.05 ± 0.07	2222 ± 35	0.278 ± 0.02	0.72

^a^ Expressed as °Brix; ^b^ Expressed as g of malic acid per 100 g fresh weight; ^c^ Expressed as mg of gallic acid per 100 g fresh weight; ^d^ Expressed as EC_50_: mg of extract per mL DPPH.

### 2.2. Chemical Characterization of the Lipophilic Extractives from A. unedo Berries

The dichloromethane extraction yields (Table 1) of ripe *A. unedo* fruits ranged between 0.72% and 1.66%. We observed that the lowest extractive yield, 0.72%, found in sample 6, is nearly half of the extractive yields obtained for the berries collected from location 1 to 5. The great discrepancy in the lipidic content observed in *A. unedo* is in agreement with previously reported data concerning other berries, namely mulberries (*Morus* spp.), which ranged from 0.14% to 0.40%. These fluctuations may be due to different environmental conditions where the species are grown [34] and also to the genetic diversity of the plants [35]. To the best of our knowledge there are no reports in the literature about lipophilic extractives yields of *A. unedo* fruits. Nevertheless, these lipophilic contents were similar to those found for other berries such as elderberries (*Sambucus nigra* L.) [36].

**Figure 1:**
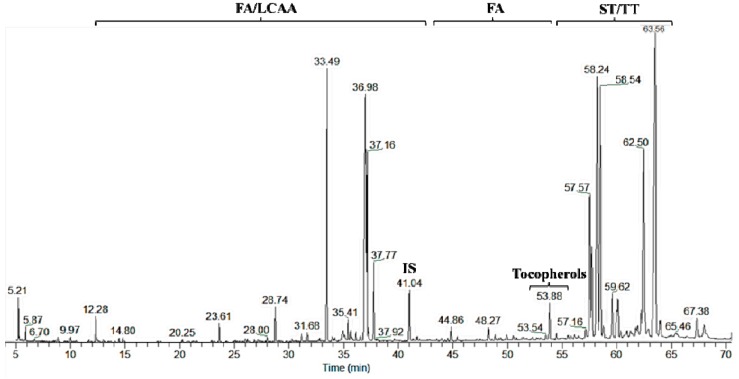
GC–MS chromatogram of the trimethylsilyl (TMS)-derivatized dichloromethane extracts of *A. unedo* berries after alkaline hydrolysis. Abbreviations: FA, fatty acids; LCAA, long chain aliphatic alcohols; IS, internal standard; ST, sterols; TT, triterpenoids.

The lipophilic fraction of *A. unedo* fruits harvested from six different locations presented similar compositions, either before or after alkaline hydrolysis. Figure 1 shows a typical GC–MS chromatogram of the *A. unedo* berries dichloromethane derivatized extract after alkaline hydrolysis.

**Figure 2:**
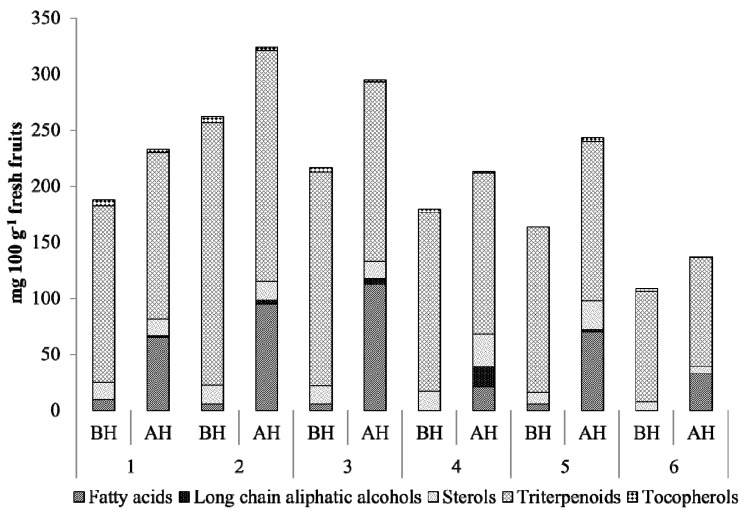
Major families of lipophilic compounds identified in *A. unedo* berries collected from six locations in Penacova (center of Portugal), before (BH) and after (AH) alkaline hydrolysis.

The GC–MS analysis allowed the identification of 41 compounds belonging to five chemical families, namely triterpenoids, sterols, fatty acids, long chain aliphatic alcohols and tocopherols. The detailed information about the individual components identified, expressed as mg of compound per 100 g of fresh berries, before and after alkaline hydrolysis, is summarized in Table 2. Triterpenoids, followed by fatty acids and sterols, were the major families of lipophilic compounds found in fruits from all locations as illustrated in Figure 2 and Table 2.

Triterpenoids represented the major family of lipophilic components of *A. unedo* berries (Table 2). This family accounted for up to 90% of the total amount of detected compounds before hydrolysis and up to 70% after hydrolysis (mainly due to the strong increment in the amount of fatty acids). The triterpenoids content ranged from 97 and 99 mg per 100 g of fresh weight after and before hydrolysis, respectively, in location 6, to 206 and 234 mg per 100 g of fresh weight after and before hydrolysis, respectively, in location 2. Ursolic acid was the major compound representing up to 39% of all identified compounds before hydrolysis and up to 25% after hydrolysis.

Other identified triterpenoids included lupeol, oleanolic acid, uvaol, olean-12-en-3β,23-diol, α- and β-amyrin and lupeol derivatives. All the triterpenoids identified in the present study have been previously described as *A. unedo* fruits lipophilic components [27], but without any quantification The total triterpenoids content of strawberry tree fruits is within the range of 100–240 mg of triterpenoids per 100 g fresh fruit, a value higher than that reported for other berries such as bilberry (*Vaccinium myrtillus* L.), which ranges between *ca*. 75 and 80 mg triterpenoids per 100 g fresh fruit [37].

Fatty acids (Table 2) represented the second major group of lipophilic compounds present in *A. unedo* fruits, corresponding to 1%–5% (1 to 10 mg per 100 g of fresh weight) of the total amount of detected lipophilic compounds in the dry extract before hydrolysis and between 10% and 38% after hydrolysis (21 to 112 mg per 100 g of fresh weight).

**Table 2:** Lipophilic composition (expressed as mg per 100 g^−1^ fresh weight) of *A. unedo* berries collected from six locations in Penacova (center of Portugal) Portugal, before (BH) and after (AH) alkaline hydrolysis. ^a^

R.t. (min)	Compound	Locations											
		1		2		3		4		5		6		
		BH	AH	BH	AH	BH	AH	BH	AH	BH	AH	BH	AH	
	**Fatty Acids**	**10**	**75**	**6**	**95**	**5**	**120**	**1**	**21**	**5**	**70**	**1**	**33**
	*Saturated*	6	19	3	20	4	28	tr	10	5	24	0	14
8.46	Hexanoic acid C6:0	nd	tr	nd	tr	nd	tr	nd	tr	nd	tr	nd	tr
11.68	Octanoic acid C8:0	nd	tr	nd	tr	nd	tr	nd	tr	nd	tr	nd	tr
14.87	Nonanoic acid C9:0	nd	tr	nd	tr	nd	tr	nd	tr	nd	tr	nd	tr
17.96	Decanoic acid C10:0	nd	tr	nd	tr	nd	tr	nd	tr	nd	tr	nd	tr
23.61	Dodecanoic acid C12:0	tr	1	tr	tr	tr	tr	tr	tr	tr	tr	tr	tr
26.94	Tridecanoic acid C13:0	nd	tr	nd	tr	nd	tr	nd	tr	nd	tr	nd	tr
28.74	Tetradecanoic acid C14:0	nd	2	nd	1	nd	2	nd	1	nd	1	nd	2
31.78	Pentadecanoic acid C15:0	nd	tr	nd	tr	nd	tr	nd	tr	nd	tr	nd	tr
33.49	Hexadecanoic acid C16:0	6	12	3	12	4	23	nd	4	nd	10	nd	9
35.64	Heptadecanoic acid C17:0	nd	tr	nd	tr	nd	tr	nd	3	nd	7	nd	tr
37.77	Octadecanoic acid C18:0	tr	5	tr	8	tr	10	tr	2	5	4	tr	3
41.73	Eicosanoic acid C20:0	nd	tr	nd	tr	nd	tr	nd	tr	nd	1	nd	tr
45.45	Docosanoic acid C22:0	tr	tr	nd	tr	tr	tr	tr	tr	tr	1	tr	tr
47.08	Tricosanoic acid C23:0	nd	tr	nd	tr	nd	tr	nd	tr	nd	tr	nd	tr
48.91	Tetracosanoic acid C24:0	tr	tr	tr	tr	tr	tr	tr	tr	tr	tr	tr	tr
	*Unsaturated*	4	46	2	75	1	85	1	11	1	45	1	19
32.68	Hexadec-9-enoic acid C16:1 ^(Δ9)^ isomer	tr	tr	tr	tr	tr	tr	tr	1	tr	tr	tr	10
32.81	Hexadec-9-enoic acid C16:1 ^(Δ9)^ isomer												
36.98	Octadeca-9,12-dienoic acid C18:2 ^(^^Δ^^9, 12)^ + Octadeca-9,12,15-trienoic acid C18:3 ^(^^Δ^^9, 12, 15)^	4	33	2	75	1	85	1	10	1	45	1	6
37.16	Octadec-9-enoic acid C18:1 ^(Δ9)^ isomer	tr	23	tr	tr	tr	tr	tr	tr	tr	tr	tr	3
37.26	Octadec-9-enoic acid C18:1 ^(Δ9)^ isomer												
	**Long Chain Aliphatic Alcohols**	**0**	**2**	**0**	**4**	**0**	**5**	**0**	**17**	**0**	**2**	**0**	**tr**
26.84	Tetradecan-1-ol	nd	tr	nd	tr	nd	tr	nd	1	nd	tr	nd
31.68	Hexadecan-1-ol	nd	tr	nd	tr	nd	1	nd	9	nd	1	nd	tr
35.41	Octadec-9-en-1-ol	nd	2	nd	4	nd	4	nd	7	nd	1	nd	tr
36.12	Octadecan-1-ol	nd	tr	nd	tr	nd	tr	nd	tr	nd	tr	nd	tr
	**Sterols**	**15**	**15**	**17**	**16**	**16**	**16**	**16**	**30**	**11**	**26**	**7**	**6**
55.09	Cholestan-3-one	nd	tr	tr	tr	tr	tr	tr	tr	tr	tr	tr	tr
55.83	Campesterol	nd	1	nd	1	nd	tr	nd	tr	nd	4	nd	tr
56.65	Stigmasterol	tr	tr	tr	tr	tr	tr	tr	tr	tr	tr	tr	tr
57.75	Sitosterol	15	14	17	16	16	16	16	30	11	21	7	6
	**Triterpenoids**	**157**	**149**	**234**	**206**	**191**	**160**	**160**	**144**	**147**	**142**	**99**	**97**
57.16	Amyrin derivative	2	tr	2	2	2	tr	2	12	1	2	1	tr
57.33	Lupenone	3	2	3	3	4	3	3	3	2	1	1	1
57.57	β-Amyrin	2	1	1	1	2	1	2	26	tr	6	1	13
58.24	α-Amyrin	29	26	36	31	32	28	36	35	24	38	37	50
58.54	Lupeol	35	35	49	44	33	30	31	3	31	43	3	tr
59.62	Lupenyl acetate	5	5	8	7	6	5	1	1	3	3	2	3
62.10	Olean-12-en-3β,23-diol	3	3	5	5	3	3	3	3	3	3	1	2
62.25	Uvaol	1	2	35	31	26	23	3	4	18	7	1	1
62.50	Oleanolic acid	24	22	2	1	2	2	17	21	1	10	10	8
63.56	Ursolic acid	54	53	93	81	81	63	62	36	63	28	42	20
	**Tocopherols**	**10**	**2**	**6**	**3**	**4**	**2**	**3**	**1**	**1**	**4**	**2**	**1**
53.88	α-Tocopherol	4	1	3	2	1	1	1	1	1	2	1	tr
54.18	γ-Tocopherol	6	2	3	1	3	1	2	tr	tr	2	1	tr
	**Total (mg 100 g^−1^ fresh fruit)**	**192**	**243**	**262**	**324**	**217**	**303**	**179**	**213**	**164**	**243**	**109**	**137**

^a^ Abbreviations: nd, not detected; tr, traces. The results are the average of the concordant values obtained for each sample (less than 5% variation between injections).

After hydrolysis, the total fatty acids content increased up to 70 times and the content of unsaturated fatty acids was found to be considerably higher than that of saturated fatty acids in all samples, with the exception of location 4. The highest ratio of unsaturated/saturated fatty acids was about 3.6 in location 2 and the lowest was about 1 in location 4. As illustrated in Figure 2 and Table 2, it can be seen that the fatty acid content of the berries harvested from location 4 is the lowest, when compared to the remaining samples. This difference can be attributed to the complex interactions between intrinsic factors, such as the genetic factors and vegetative stage of the plant, and extrinsic factors such as type of soil, microclimatic conditions (level of radiation, temperature, wind exposure and water availability) and geographic position [35].

The identified fatty acids ranged from hexanoic (C6:0) to tetracosanoic acids (C24:0), including six unsaturated structures (*cis-* and *trans-*C16:1 and C18:1, C18:2 and C18:3). Hexadecanoic acid (C16:0) was the most abundant saturated fatty acid in *A. unedo* fruits with the highest content observed in location 3, with 16 mg per 100 g of fresh weight after hydrolysis. Octadeca-9,12-dienoic (ω-6) and octadeca-9,12,15-trienoic (ω-3) acids were the major unsaturated fatty acids reaching up to 85 mg per 100 g of fresh weight in location 3 after hydrolysis. These were followed by *cis*- and *trans*-octadec-9-enoic acids and *cis*- and *trans*-hexadec-9-enoic acids. The compounds identified in the present study have already been reported in *A. unedo* fruits however, only the contents of hexadecanoic, octadec-9-enoic, octadeca-9,12-dienoic and octadeca-9,12,15-trienoic acids were reported [15,18,30]. Previous studies revealed that octadeca-9,12-dienoic and octadeca-9,12,15-trienoic acids accounted for 92.1 ± 6.6 mg and 83.5 ± 16.3 mg per 100 g, followed by hexadecanoic and octadec-9-enoic acids which accounted for 50.01 ± 1.0 and 39.9 ± 9.2 mg per 100 g fresh weight respectively [30]. In this study, the octadeca-9,12-dienoic and octadeca-9,12,15-trienoic acids content represented about 120 mg per 100 g fresh fruit, about 23 mg of octadec-9-enoic acid and 24 mg of hexadecanoic acid (Table 2). These results were in agreement with previously reported data on *A. unedo* berries: unsaturated fatty acids were predominant over the saturated fatty acids, with octadeca-9,12-dienoic and octadeca-9,12,15-trienoic acids as the major compounds after hydrolysis [15,30].

The sterols found in *A. unedo* include cholestan-3-one, campesterol, stigmasterol and β-sitosterol (Table 2). These phytosterols represented up to 9% of the total extract before hydrolysis and up to 14% after hydrolysis (6–30 mg per 100 g of fresh weight). β-sitosterol was the main component of this family, representing between 82% and 100% of total sterols content (6–30 mg per 100 g of fresh weight). With the exception of campesterol all the sterols identified in the present study have already been reported in the literature as *A. unedo* berries components [27], but their quantification was not reported before. The sterols content of strawberry tree fruits reported here (Table 2) is similar to the values reported for other berries, such as bilberry (*Vaccinium myrtillus* L.), with *ca*. 18 and 23 mg sterols per 100 g fresh fruit [37], and higher than that reported for sea-buckthorn (*Hippophae rhamnoides* L.) (0.33 to 0.53 mg per 100 g of fresh weight) [38].

Long-chain aliphatic alcohols were detected in quite low amounts after alkaline hydrolysis, representing about 1%–8% of the total lipophilic extractives of ripe *A. unedo* fruits (ranged from 2 to 17 mg per 100 g of fresh weight, for locations 2 and 5, and 4, respectively). No aliphatic alcohols have been detected before hydrolysis. Octadec-9-en-1-ol was the most abundant compound identified, representing 43%–100% of the total long-chain aliphatic alcohols.

Tocopherols (α- and γ-tocopherols) were detected as the minor class of compounds in *A. unedo* berries. The highest tocopherol content (10 mg per 100 g of fresh weight) was found in location 1, before hydrolysis, and after hydrolysis in location 5 with 4 mg per 100 g of fresh weight of berries. The content of tocopherols found in strawberry tree fruits was higher than that reported in literature for the same fruits, with 0.023–9.4 mg per 100 g fresh weight [7,18].

## 3. Experimental Section

### 3.1. Chemicals

Dichloromethane (≥99% purity), methanol (≥99.8% purity) and sodium hydroxide (≥97% purity) were supplied by Fischer Scientific (Pittsburgh, PA, USA). Hydrochloric acid (≥37%) was purchased from Fluka Chemie (Madrid, Spain). Tetracosane (99% purity), *N*,*O*-bis(trimethylsilyl)trifluoroacetamide (99% purity), trimethylchlorosilane (99% purity), hexadecanoic acid (≥99% purity), nonadecan-1-ol (99% purity), cholesterol (99% purity), ursolic acid (≥90% purity), oleanolic acid (≥97% purity), and betulinic acid (≥98% purity) were obtained from Sigma Chemicals Co. (Madrid, Spain). Pyridine (≥99.5% purity) was purchased from Panreac (Castellar del Vallès, Spain).

### 3.2. Samples

*A. unedo* berries were supplied by Medronhalva Lda., from São Pedro de Alva, in Penacova, Portugal. The wild fruits were harvested from 6 locations (Table 1) in order to acquire a representative sampling, reflecting the natural variability of the berries from that region.

*A. unedo* berries (around 500 g for each sample) were hand-harvested on-site at a mature stage, in 11 December 2013. The mature stage was established based on the following parameters: total soluble solids (expressed as °Brix, which should be at least 16.5 [31]), titratable acidity (should be at least 0.4 g of malic acid per 100 g fresh fruit [31]) and also on fruit’s colour (homogenous red-scarlet pigmentation). After harvesting, the fruits were refrigerated, transported to the laboratory and stored at −20 °C until analysis.

### 3.3. General Chemical Characterization

The total soluble solids content, titratable acidity and pH determination was performed as described by Ruiz-Rodríguez *et al.* [22]. The total soluble solids content was determined using a hand-held refractometer (Atago) directly in the fruits pulp. pH was measured using a Crison micropH 2000 pH-meter over an homogenized sample (obtained from the fruits manually crushed) 1/4 (*w*/*v*) in distilled water. TA was determined by titration with 0.1 M NaOH until pH of 8.1 was reached and was expressed as g malic acid per100 g fresh weight.

Prior to the total phenolic content and the antioxidant activity determination, the samples were prepared accordingly to *Santos et al.* [39]. *A. unedo* berries were freeze-dried using a VirTis BenchTop K (SP Industries, New York, NY, USA), and the water content determined (105 °C/8 h). Nearly 15 g (±0.1) of each sample were accurately weighed and ground prior to extraction and submitted to a Soxhlet extraction with dichloromethane for 6 h to remove the lipophilic fraction. The solid residue was suspended (mass/volume 1:100) in a methanol/water/acetic acid mixture, 49.5:49.5:1 at room temperature for 24 h under constant stirring to extract the phenolic compounds. The suspension was then filtered, methanol removed by low-pressure evaporation, and the extract freeze-dried. All extractions were performed in triplicate.

The total phenolic content of the samples was determined by the Folin-Ciocalteu method as described by Singleton [40]. The method is based on the addition of 500 μL of distilled water to 125 μL of the diluted sample (the previously mentioned extract) and 125 μL of Folin-Ciocalteu reagent. The mixture is kept for 5 min and then 1.25 mL of Na_2_CO_3_ (75 g·L^−1^) and 1 mL of distilled water are added. The solutions were kept for 30 min and then the absorbance was measured at λ = 760 nm. All the measurements were made in triplicate, using three aliquots of each extract and the average value was calculated in each case. The total phenolic content was calculated as gallic acid equivalents from the calibration curve of gallic acid standard solutions (50–250 mg·L^−1^; calibration curve: *y* = 0.0045*x* + 0.049; *r^2^* = 0.9989) and expressed as mg of gallic acid equivalent (GAE) per 100 g fresh weight [39].

The antioxidant activity was determined by the DPPH method [41]. Briefly, 3.9 mL of DPPH solution was added to 0.1 mL of sample containing different concentrations (0.313, 0.625, 1.25, 2.5 and 5 mg extract per mL of solution). The mixture was kept for 30 min in the dark. The absorbance was measured at 515 nm, in a Perkin Elmer Lambda 35 UV-Vis Spectrometer and compared with a control sample. All measurements were made in triplicate, using three aliquots of each extract and the average value was calculated in each case. The antioxidant activity was expressed as EC_50_, mg of dry extract that reduces DPPH in 50% [39]. EC_50_ was calculated by: (Abs control − Abs sample)/Abs control × 100 and were determined from the plotted graphs of EC_50_ against the concentration of the extracts.

For all the above reported methods, three independent aliquots of each sample were analysed.

### 3.4. Lipophilic Compounds Determination

#### 3.4.1. Extraction

The lipophilic extracts were obtained as aforementioned in Section 3.3, prior to the extraction of the phenolic fraction. To the extraction of the lipophilic fraction, dichloromethane was chosen because it is a fairly specific solvent for lipophilic extractives for analytical purposes [42].

#### 3.4.2. Alkaline Hydrolysis

About 20 mg (±0.1) of each lipophilic extract was accurately weighed and dissolved in 10 mL of 1 M NaOH in 50% aqueous methanol. The mixture was heated at 100 °C, under a nitrogen atmosphere, for 1 h. The reaction mixture was cooled, acidified with 1 M HCl to pH ≈ 2, and then extracted three times with dichloromethane, and the solvent was evaporated to dryness [42]. The alkaline hydrolysis reaction was performed to detect indirectly esterified compounds, e.g., triglycerides, steryl esters, *etc.*

#### 3.4.3. GC–MS Analysis

Before GC–MS analysis, nearly 20 mg (±0.1) of each DCM extract solid residue was accurately weighed and converted into trimethylsilyl (TMS) derivatives according to a previously optimized methodology [43]. Briefly, each sample was dissolved in 250 μL of pyridine containing 1 mg of tetracosane (internal standard), and compounds with hydroxyl and carboxyl groups were converted into trimethylsilyl ethers and esters, respectively, by adding 250 μL of *N*,*O*-bis(trimethylsilyl)-trifluoroacetamide and 50 μL of trimethylchlorosilane. The mixture was maintained at 70 °C for 30 min.

GC–MS analyses were performed using a Trace Gas Chromatograph 2000 Series equipped with a Thermo Scientific DSQ II single-quadropole mass spectrometer (Waltham, MA, USA). Separation of compounds was carried out in a DB-1 J&W capillary column (30 m × 0.32 mm inner diameter, 0.25 μm film thickness, Agilent Technologies, Santa Clara, CA, USA) using helium as the carrier gas (35 cm·s^−1^). The chromatographic conditions were as follows: initial temperature, 80 °C for 5 min, temperature rate, 4 °C·min^−1^ up to 260 °C, 2 °C·min^−1^ up to 285 °C, then maintained for 10 min; injector temperature, 250 °C; transfer-line temperature, 290 °C, split ratio, 1:50. The mass spectrometer was operated in the electron impact (EI) mode with electron impact energy of 70 eV, and data were collected at a rate of 1 scan·s^−1^ over a range of *m*/*z* 33–700. The ion source was kept at 250 °C. Data acquisition was carried out with the Xcalibur data system (ThermoFinnigan, San Jose, CA, USA).

Chromatographic peaks were identified as TMS derivatives by comparing their mass spectra with the equipment mass spectral library (Wiley-NIST Mass Spectral Library, Oxford, UK, 1999), with literature MS fragmentation data [43,44,45,46] and also by injection of standards. For semi-quantitative analysis, GC–MS was calibrated with hexadecanoic acid for fatty acids, nonadecan-1-ol for long chain aliphatic alcohols, cholesterol for phytosterols, ursolic, oleanolic and betulinic acids for the corresponding compounds relative to tetracosane, used as internal standard as described in detail in previous studies [44,45]. The respective response factors needed to obtain correct quantification of the peak areas were calculated as the average of four GC–MS runs. For tocopherol the response factor of sterols was used. The compound contents are expressed as mg per 100 g of fresh weight of fruit. For each extract, two independent aliquots before alkaline hydrolysis, and another two aliquots after alkaline hydrolysis were prepared. Each aliquot was injected in triplicate. The results of Figure 2 and Table 2 are the average of the concordant values obtained for each sample (less than 5% variation between injections of the same aliquot).

## 4. Conclusions

The present work represents a detailed phytochemical study about the lipophilic fraction from ripe *A. unedo* berries, being the berries harvested from six locations from Penacova (center of Portugal). Fatty acids, triterpenoids, sterols, long-chain aliphatic alcohols and tocopherols were the main chemical families under study, with triterpenoids as the predominant compounds, followed by fatty acids and sterols. Long-chain aliphatic alcohols and tocopherols were identified as minor components. Among the 41 compounds identified, ursolic acid, lupeol, linoleic and α-linolenic acids and β-sitosterol were the main constituents. To the best of our knowledge, long chain fatty alcohols, as well as campesterol, were reported for the first time as components of strawberry tree berries and a comprehensive quantification of several lipophilic components was accomplished also for the first time. The present study also unveiled the *A. unedo* berries as a source of ω-3 and ω-6 fatty acids, phytosterols and tocopherols, compounds with well-established beneficial effects in human health. This may be useful to value *A. unedo* fruits as sources of valuable phytochemicals and enhance future applications with nutritional, pharmacological or cosmetic purposes. Furthermore, this information can also be relevant for the valorization of residues from *A. unedo* alcoholic beverages distillery. A high variability in the lipophilic content of wild *A. unedo* berries collected from the six locations was observed, therefore it will be important to study the effect of ripening, season, genotype, harvesting and processing in order to understand the profile of variation of the lipophilic composition of *A. unedo* berries. Finally, the full understanding of these berries nutraceutical potential also require a systematic analysis of other fractions as for example phenolic compounds, which will be reported soon.
